# Toxic encephalopathy due to mercury: A rare case report and literature review

**DOI:** 10.1097/MD.0000000000044037

**Published:** 2025-09-12

**Authors:** Menghong Zou, Junfeng Li, Hongchao Yao, Qionglan Dong, Qiang Zeng, Qibing Tang, Jie Zhang, Hongwei Li, Jisheng Wang

**Affiliations:** a Department of Radiology, The Third Hospital of Mianyang (Sichuan Mental Health Center), Mianyang, Sichuan Province, People’s Republic of China; b School of Pharmacy, Southwest Medical University, Luzhou, Sichuan Province, People’s Republic of China; c Intensive Care Unit, The Third Hospital of Mianyang (Sichuan Mental Health Center), Mianyang, Sichuan Province, People’s Republic of China; d Department of Respiratory Medicine, The Third Hospital of Mianyang (Sichuan Mental Health Center), Mianyang, Sichuan Province, People’s Republic of China; e Department of Pharmacy, The Third Hospital of Mianyang (Sichuan Mental Health Center), Mianyang, Sichuan Province, People’s Republic of China.

**Keywords:** case report, imaging features, mercury toxic encephalopathy, neurology, radiology

## Abstract

**Rationale:**

Mercury toxic encephalopathy is uncommonly encountered in clinical practice.

**Patient concerns:**

The aim of presenting this case is to increase the awareness of this disease, especially regarding its radiological presentation.

**Diagnoses:**

Toxic encephalopathy due to mercury.

**Interventions:**

The medical team administered sodium dimercaptopropane sulfonate as a mercury antagonist and treated with plasma purification and massive hydration to promote mercury excretion.

**Outcomes:**

Following admission, the patient underwent an magnetic resonance imaging (MRI) examination. The results revealed symmetrical alterations in the brain parenchyma (including both cerebrum and cerebellum), characterized by hypoperfusion on MRI perfusion imaging. The patient died after a series of lifesaving measures.

**Lessons:**

Mercury poisoning encephalopathy is a rare disorder for which currently established diagnostic criteria are lacking. On brain MRI scans, it manifests as symmetric cerebral edema, involving the parenchyma of both the cerebrum and cerebellum. It is important to question patients carefully about any history of exposure to mercury-containing drugs.

## 1. Introduction

Mercury toxic encephalopathy, a relatively rare form of toxic encephalopathy, presents clinically with symptoms such as headache, depression, dizziness, and impaired consciousness.^[1]^ Characteristic imaging findings include bilateral symmetrical damage affecting the cerebral hemispheres, basal ganglia, and cerebellar hemispheres. Treatment is centered on mercury chelation therapy. While mild cases diagnosed and treated promptly often have a good prognosis, severe cases typically carry a poor prognosis.

## 2. Case presentation

A 34-year-old man with a >10-year history of psoriasis presented to our hospital with a 1-day history of progressive dyspnea, sore throat, and impaired consciousness.

Two days prior to admission, he had inhaled a mercury-containing traditional Chinese medicine for psoriasis. Shortly after, he developed a sore throat, dyspnea, and began expectorating small amounts of white sputum, without neurological, or digestive symptoms. One day prior to admission, his dyspnea worsened significantly, and he developed perioral blisters and dry lips. He subsequently experienced progressive altered consciousness culminating in respiratory arrest, necessitating emergency transfer to our facility by our medical team.

Admission investigations:

- Toxicology: Blood mercury: 201.8 μg/L; Urine mercury: >500 μmol/L.
- Cardiac markers: Troponin I: 1703.73 ng/L; Myoglobin: >1000.00 ng/ml.
- Cardiac enzymes: aspartate aminotransferase: 1201 U/L, lactic dehydrogenase: 2290 U/L, hydroxybutyrate dehydrogenase: 848 U/L, creatine kinase: 2537 U/L.
- Arterial blood gas: pH: 7.49, pCO₂: 51 mm Hg, pO₂: 120 mm Hg, HCO₃⁻: 35.4 mmol/L, Lactate: 2.6 mmol/L, PaO₂/FiO₂ ratio: 267 (Table 1).

**Table 1:** Patient admission blood indices and blood gas analysis results.

Form	Value
Blood mercury	201.8 μg/L
Urine mercury level	>500 μmol/L
Blood troponin I concentration	1703.73 ng/L
Blood myoglobin concentration	1000.00 ng/mL
AST	1201 U/L
LDH	2290 U/L
HBDH	848 U/L
CK	2537 U/L
pH	7.49
Carbon dioxide	51
Partial pressure of oxygen	120
Bicarbonate	35.4
Mammary gland	2.6
Oxygenation index	2.67

AST = aspartate aminotransferase, CK = creatine kinase, HBDH = hydroxybutyrate dehydrogenase, LDH = lactic dehydrogenase.

**Figure 1.**
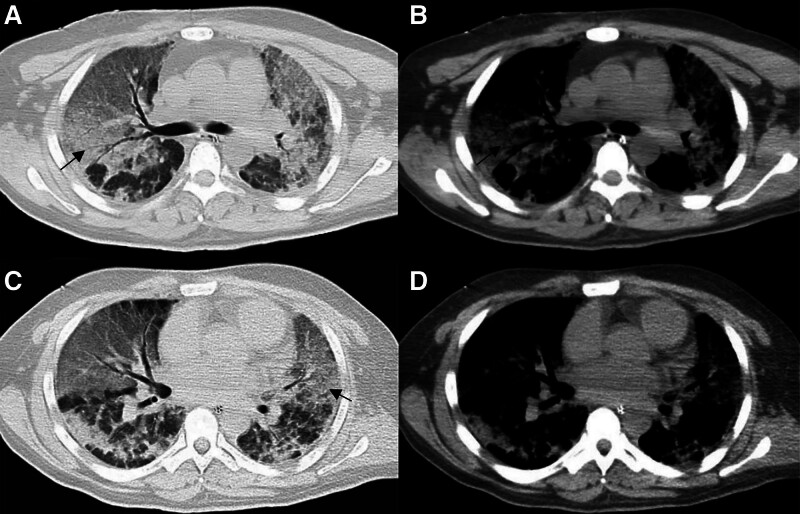
Computed tomography of the chest. (A and C) Chest computed tomography scans obtained with lung window settings. (B and D) Mediastinal window images of the chest computed tomography scan from the patient. The black arrows demonstrate extensive interstitial changes in both lungs.

**Figure 2.**
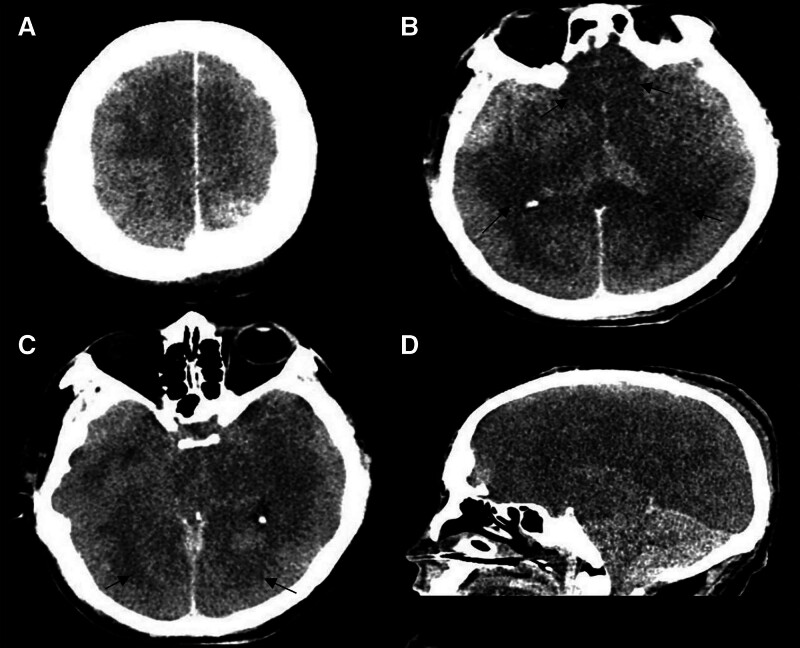
Computed tomography of the head. (A–C) Axial images from the patient’s noncontrast head computed tomography scan, displayed in brain windows. (D) The patient’s cranial computed tomography brain window images in the sagittal view. The black arrow demonstrates symmetric involvement of the brain parenchyma.

**Figure 3.**
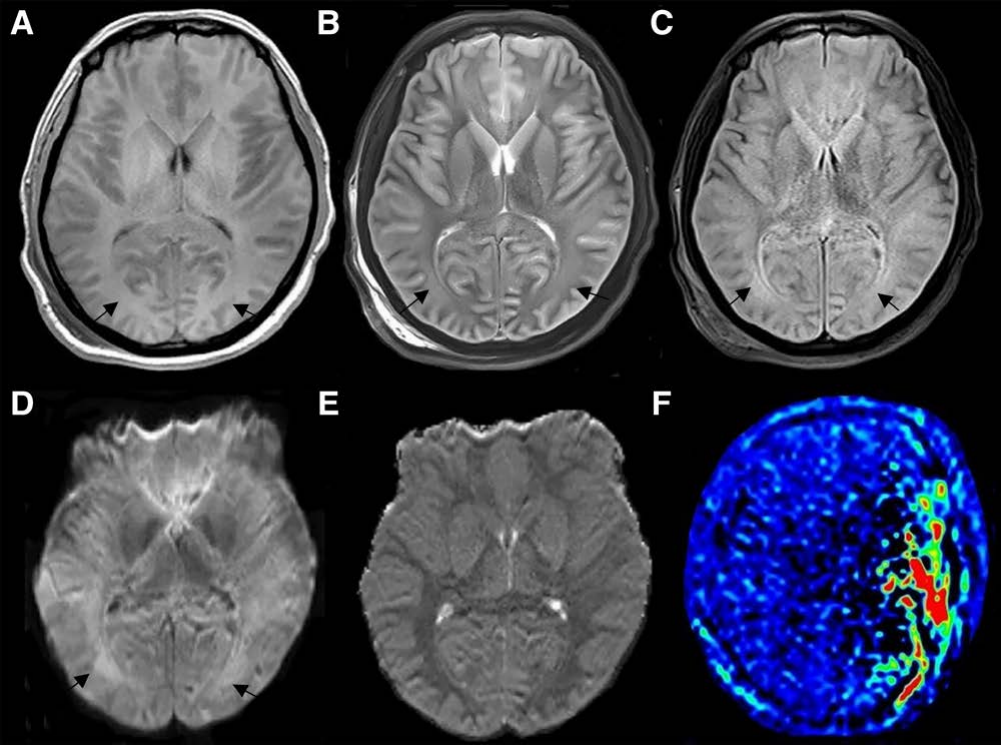
MRI of the head. Symmetrical distribution of low-signal lesions in T1 sequences (A). Low-signal lesions in T2 sequences (B). And a high FLAIR sequence signal (C). Restricted symmetric diffusion in both cerebral hemispheres (D, E). And asymmetrical reduced perfusion of brain tissue (F). The black arrow demonstrates symmetric involvement of the brain parenchyma. FLAIR = fluid-attenuated inversion recovery, MRI = magnetic resonance imaging

Imaging:

- Chest computed tomography (CT): Revealed diffuse aspiration pneumonia bilaterally (Fig. 1).
- Head CT (SOMATOM Definition AS+, 5 mm slice/interval, 120 kV, 150 mA): Showed diffuse cerebral edema and symmetrical widespread hypodensities in both hemispheres (Fig. 2).
- Brain magnetic resonance imaging (MRI; Siemens Skyra 3.0T; T1: low-signal, T2: high signal lesions symmetrically; DWI: restricted diffusion; arterial spin labeling (ASL): globally reduced perfusion – TE/TR/FA: 8 ms/18 ms/20°; ASL: TE/TR/TI/slice: 11 ms/2500 ms/700 ms/6 mm): Confirmed diffuse cerebral edema and bilateral symmetrical lesions. DWI demonstrated restricted diffusion, and ASL indicated globally reduced cerebral perfusion (Fig. 3).

Diagnosis and management: Based on the history of mercury inhalation, significantly elevated mercury levels, and characteristic bilateral symmetrical hemispheric lesions, a diagnosis of mercury toxic encephalopathy was made. Treatment included intravenous sodium dimercaptopropane sulfonate (DMPS) chelation therapy, plasma purification, and aggressive hydration to promote mercury elimination.

## 3. Result

Serial monitoring showed decreasing blood mercury levels. However, cardiac enzyme levels remained persistently elevated. Despite intensive supportive care, the patient died 7 days after admission.

The patient was admitted and underwent chest CT, revealing diffuse bilateral aspiration pneumonia. Head CT demonstrated diffuse cerebral edema with symmetrical bilateral hypodense lesions throughout both hemispheres. Subsequent brain MRI confirmed diffuse cerebral edema and revealed symmetrically distributed lesions exhibiting:

- Mild T1 hypointensity,
- Mild T2 hyperintensity,
- Restricted diffusion on DWI,
- ASL perfusion imaging indicated globally reduced cerebral perfusion.

## 4. Discussion

Although an isolated case, the characteristics demonstrated in the central nervous system (CNS) imaging findings stimulated profound reflection on the pathological mechanisms, key diagnostic points, and differential diagnoses of mercury poisoning encephalopathy. Mercury is that mercury ions (Hg²⁺) exert their whitening effect primarily through irreversible inhibition of tyrosinase. Hg²⁺ covalently binds to sulfhydryl groups (–SH) of cysteine residues in the enzyme’s active site displacing essential copper cofactors (Cu²⁺) and inducing conformational changes that abrogate catalytic function. This disrupts the oxidation of tyrosine to dopaquinone, thereby suppressing melanin synthesis and reducing epidermal pigmentation.^[2]^ In China, mercury-containing compounds are occasionally used in unconventional treatments for skin conditions, with some folk remedies incorporating cinnabar (mercury sulfide). When ingested, mercury sulfide undergoes metabolic conversion in the stomach to form mercuric chloride, which is readily absorbed through the gastrointestinal tract and may induce mercury poisoning.^[3]^ Mercury toxicity primarily targets both the central and peripheral nervous systems, though the affected sites and severity of neurological impairment vary significantly, resulting in diverse clinical manifestations. Recent reports document cases of iatrogenic mercury poisoning linked to inappropriate dermatological treatments.^[4]^ Notably, some patients self-administer increased dosages without medical supervision in an attempt to enhance therapeutic efficacy, thereby escalating poisoning risks. In the present case, the patient developed mercury poisoning following substantial inhalation of mercury-based preparations for psoriasis management. Elevated blood and urine mercury levels confirmed the diagnosis.

Given the patient’s diagnosis of severe mercury poisoning complicated by toxic encephalopathy, chelation therapy combined with blood purification was immediately initiated. Sodium DMPS was employed as the first-line chelating agent. Its mechanism of action involves binding circulating free heavy metal ions to form water-soluble complexes, thereby accelerating their renal excretion. Concurrently, plasma purification (specifically, plasma exchange, or double filtration plasmapheresis) was implemented. This modality aims to rapidly remove heavy metals bound to plasma proteins and inflammatory mediators, mitigating target organ damage and ameliorating internal milieu disturbances. DMPS administration emphasizes early and adequate dosing. Literature indicates that initiating chelation therapy within the 24- to 48-hour window post-intoxication significantly enhances heavy metal clearance efficiency and improves prognosis.^[5,6]^ Particularly for patients already manifesting severe complications, delayed treatment substantially increases the risk of irreversible organ damage. In this case, the interval between the onset of intoxication and the commencement of systematic treatment upon hospitalization was approximately 48 hours. This may have exceeded the optimal therapeutic window. Consequently, the patient likely presented with a high tissue burden of heavy metal and widespread preexisting damage, which was a significant contributing factor to the suboptimal therapeutic response and poor prognosis. Despite combined therapy with DMPS chelation and plasma purification, the patient’s prognosis remained extremely poor. This outcome underscores the high mortality associated with severe mercury poisoning and the considerable therapeutic challenges involved. This case serves as a critical reminder that for suspected heavy metal poisoning, especially in patients presenting with severe symptoms, time is of critical essence. Clinicians must maintain a high index of suspicion, strive for early diagnosis, and administer adequate doses of an effective chelator within the optimal therapeutic window (24–48 hours post-exposure). Plasma purification can serve as an adjunctive measure in critically ill patients to remove protein-bound toxins and stabilize the internal environment. However, its application requires individualized risk-benefit assessment. Importantly, even with aggressive treatment, established multi-organ dysfunction syndrome frequently leads to adverse outcomes. Future research is warranted to explore more effective early intervention strategies and develop comprehensive management approaches specifically tailored for critically ill patients with severe heavy metal poisoning.

Current research confirms that mercury and its compounds cause chronic peripheral nerve damage, though the underlying pathological mechanisms remain unclear. Watkins and Maier^[7]^ reported that mercury-induced chronic damage primarily manifests as neuropathic pain. This pain is closely linked to astrocyte activation in peripheral nerves, triggering the release of neuroactive substances and proinflammatory cytokines that act on nociceptive neurons in the spinal cord’s dorsal horn. McRill et al^[8]^ demonstrated that mercury poisoning affecting the CNS commonly presents as headaches, depression, dizziness, anxiety, generalized weakness, mental excitability, and subsequent joint/limb pain. Mercury ions (Hg^2+^), highly lipophilic and diffusible, readily cross the blood-brain barrier. Upon entering the brain, elemental mercury oxidizes to Hg^2+^, binds to tissue proteins, accumulates, and induces headaches, and cytotoxic cerebral edema. In the present case, the patient inhaled a mercury-containing medication for psoriasis. Elemental mercury is primarily absorbed via inhalation; its high lipid solubility facilitates BBB penetration, making pulmonary manifestations (e.g., interstitial pneumonitis) and CNS disorders typical of its toxicity. Classically affected CNS regions include the cerebral cortex (pyramidal cells, astrocytes) and cerebellum (Purkinje cells, granule cells, deep cerebellar nuclei).^[9,10]^ MRI revealed symmetrical T1 hypointense and T2 hyperintense lesions in bilateral cerebral and cerebellar hemispheres, with fluid-attenuated inversion recovery hyperintensity, indicating extensive mercury-induced toxic edema. Cytotoxic edema arises from brain tissue hypoxia and impaired cell membrane function, disrupting Na^+^/K^+^/Ca^2+^ pumps and reducing ASL perfusion due to diminished cerebral blood flow.^[11,12]^ DWI sequences help determine lesion acuity: acute-phase lesions show high/isointense signals (reflecting cytotoxic/vasogenic edema), while chronic-phase lesions become hypointense, indicating irreversible changes like cell necrosis/apoptosis.

Mercury toxic encephalopathy is a rare toxic disease for which uniform diagnostic criteria are lacking. Several authors and expert groups have proposed diagnostic frameworks or emphasized critical components.^[13–15]^ While not universally adopted, these proposals generally converge on the necessity of: A compelling history of mercury exposure sufficient to cause neurological damage and a constellation of neurological/neuropsychiatric symptoms plausibly linked to mercury toxicity. Careful exclusion of other neurological diagnoses. Supportive evidence from biomarkers or neuroimaging may strengthen the diagnosis but is often not definitive alone. Establishing a diagnosis therefore relies heavily on clinical judgment based on this synthesis of information. This approach aligns with consensus on mercury neurotoxicity assessment. In the present case, the patient inhaled mercury-containing drugs for psoriasis, which clinically supported the diagnosis of mercury toxic encephalopathy. The disease also needs to be distinguished from carbon monoxide (CO) toxic encephalopathy and acute epidemic encephalitis B in MRI manifestations. CO toxicity is the formation of carboxyhemoglobin by competitive binding of hemoglobin to CO inhaled by the organism, which in turn reduces the oxygen-carrying capacity of hemoglobin and the partial pressure of oxygen in tissues, resulting in hypoxia. The pathological mechanism of CO toxic encephalopathy is cerebral vasospasm accompanied by extensive ischemia, hemorrhage and edema of brain tissue. The characteristic manifestation of CO toxic encephalopathy is bilateral symmetrical necrosis of the pallidum, which may appear within 7 to 14 days after the onset of the disease, involving different parts of the cerebral cortex, the cerebellum, the hippocampus, the amygdala, the corpus callosum and the conductive lobes; most of these patients have a corresponding history of exposure to CO. Acute epidemic encephalitis B occurs mostly in children and has an epidemiological characteristic of occurring in the summer and autumn. Clinical symptoms of acute epidemic encephalitis B include high fever accompanied by headache and vomiting. Epidemic encephalitis B virus widely affects the CNS, with the cerebral cortex, basal nuclei and thalamus being the most affected, causing meningeal congestion, cerebral edema, and the necrosis of neuronal cells. Acute epidemic encephalitis B differs from mercury toxic encephalopathy in that bilateral thalamic hemorrhagic lesions can be seen in the former, which has a high degree of specificity (Table 2).

**Table 2:** Key diagnostic features distinguishing mercury-induced encephalopathy from common neurological disorders.

	Mercury toxic encephalopathy	CO toxic encephalopathy	Acute epidemic encephalitis B
Cause of disease	Mercury intake	Inhaling a large amount of CO	Infection with Japanese encephalitis virus
Pathology	Mercury ions cross the blood-brain barrier and bind to tissue proteins after entering the brain	Cerebral vasospasm accompanied by extensive ischemia, hemorrhage, and brain tissue edema	Meningeal congestion, cerebral edema, and neuronal cell necrosis
Symptom	No specificity, may cause epilepsy, dizziness, headache, vomiting, etc	Coma, cyanosis of lips	High fever accompanied by headache and vomiting
Imaging features	Widespread involvement of brain parenchyma, multiple symmetrical imaging changes, and reduced cerebral perfusion	Bilateral symmetrical lesions of the thalamus are common and can involve the cerebral cortex, cerebellum, hippocampus, and amygdala, leading to delayed encephalopathy	The cerebral cortex, basal ganglia, and thalamus have the greatest impact, and thalamic hemorrhage may occur

CO = carbon monoxide.

This case report has the following limitations. Firstly, it is a single-case study, lacking the inclusion of a larger cohort for observation. Secondly, the patient succumbed despite aggressive treatment after admission, precluding the acquisition of posttreatment imaging for comparative analysis.

## 4. Conclusion

Mercury toxic encephalopathy is a rare toxic disease for which uniform diagnostic criteria are lacking. It is important to question patients carefully about any history of exposure to mercury-containing drugs. At the same time, patients with acute mercury poisoning tend to have inhalation poisoning, so often patients will have interstitial changes in the lungs. This is the key feature that distinguishes mercury poisoning from co-poisoning and hypoxic-ischemic encephalopathy. At the same time, the mechanism of undifferentiated involvement of cerebral and cerebellar tissues in mercury poisoning requires further in-depth study. While early intervention is crucial in mitigating the devastating effects of mercury poisoning, as demonstrated in this case, the paramount importance of prevention, particularly through targeted education regarding the risks associated with certain traditional and complementary medicines, cannot be overstated.

## Author contributions

**Conceptualization:** Menghong Zou, Junfeng Li, Qionglan Dong, Qibing Tang, Jie Zhang, Hongwei Li.

**Data curation:** Menghong Zou, Junfeng Li, Jie Zhang, Hongwei Li.

**Formal analysis:** Menghong Zou, Hongwei Li.

**Funding acquisition:** Junfeng Li.

**Investigation:** Menghong Zou, Jie Zhang, Hongwei Li, Jisheng Wang.

**Methodology:** Menghong Zou, Junfeng Li, Hongchao Yao, Hongwei Li, Jisheng Wang.

**Project administration:** Menghong Zou, Hongchao Yao, Jisheng Wang.

**Resources:** Menghong Zou, Hongchao Yao, Qiang Zeng, Qibing Tang, Jisheng Wang.

**Software:** Menghong Zou.

**Supervision:** Menghong Zou, Hongchao Yao, Qiang Zeng.

**Validation:** Menghong Zou, Qionglan Dong, Qiang Zeng.

**Visualization:** Menghong Zou, Qionglan Dong, Qiang Zeng.

**Writing – original draft:** Menghong Zou, Qionglan Dong, Hongwei Li.

**Writing – review & editing:** Menghong Zou.
